# Parameter Estimation of Fractional-Order Chaotic Systems by Using Quantum Parallel Particle Swarm Optimization Algorithm

**DOI:** 10.1371/journal.pone.0114910

**Published:** 2015-01-20

**Authors:** Yu Huang, Feng Guo, Yongling Li, Yufeng Liu

**Affiliations:** 1 Hebei Engineering Research Center of Simulation & Optimized Control for Power Generation, North China Electric Power University, Baoding, China; 2 Cognitive Science Department, School of Information Science and Engineering, Xiamen University, Xiamen, China; 3 State Key Laboratory of Power Systems, Department of Thermal Engineering, Tsinghua University, Beijing, China; University of Chinese Academy of Sciences, CHINA

## Abstract

Parameter estimation for fractional-order chaotic systems is an important issue in fractional-order chaotic control and synchronization and could be essentially formulated as a multidimensional optimization problem. A novel algorithm called quantum parallel particle swarm optimization (QPPSO) is proposed to solve the parameter estimation for fractional-order chaotic systems. The parallel characteristic of quantum computing is used in QPPSO. This characteristic increases the calculation of each generation exponentially. The behavior of particles in quantum space is restrained by the quantum evolution equation, which consists of the current rotation angle, individual optimal quantum rotation angle, and global optimal quantum rotation angle. Numerical simulation based on several typical fractional-order systems and comparisons with some typical existing algorithms show the effectiveness and efficiency of the proposed algorithm.

## Introduction

As an important concept in nonlinear science, chaos is characterized by unstable dynamic behavior with sensitive dependence on initial conditions and includes infinite unstable periodic motions. The control and synchronization of chaotic systems have gained considerable attention in recent years [1–5]. Furthermore, several researchers have recently directed increasing interest toward the chaotic behavior of fractional-order dynamical systems [6–12]. However, considerable research on the behavior of fractional-order dynamical systems requires the parameters of the fractional-order chaotic system to be determined in advance. Unfortunately, these parameters are usually unknown. In the past decades, numerous methods were proposed to solve the parameter estimation of integer-order chaotic systems. Comparatively, little attention has been devoted to the parameter estimation of fractional-order chaotic systems.

In this study, we preliminarily focus on the parameter estimation problem of fractional-order chaotic systems; those problem can be formulated as a multidimensional optimization problem. To solve this multidimensional optimization problem, a novel algorithm called quantum parallel particle swarm optimization (QPPSO) is proposed. The parallel characteristic of quantum computing is used to improve the ergodicity of QPPSO. Moreover, quantum states are updated by a new quantum evolution equation, which is constituted by the current rotation angle, individual optimal quantum rotation angle, and global optimal quantum rotation angle. The performance of QPPSO in solving the parameter estimation problem of fractional-order chaotic systems is investigated through a comparison with those of other evolutionary optimization algorithms.

The remainder of this paper is organized as follows. A brief review of relevant work on the chaotic behavior of fractional-order dynamical systems and the parameter estimation of chaotic systems is presented in Section 2. Parameter estimation for fractional-order chaotic systems from the viewpoint of optimization is formulated in Section 3. A discussion on QPPSO after a brief introduction of the quantum parallelism character is made in Section 4. Numerical simulation results based on several typical fractional-order chaotic systems and comparisons with a few existing approaches are provided in Section 5. Finally, the conclusions and a brief summary of the results are presented in Section 6.

## Literature Review

Considerable research has been done on the chaotic behavior of fractional-order dynamical systems. The chaotic behavior of the fractional-order Lorenz system was studied in [13], in which the authors determined that the system with *Σ* < 3, where *Σ* is defined as the sum of the orders of all involved derivatives, can exhibit chaotic behavior. In [14], the chaos and hyperchaos of fractional-order Rössler equations were studied. In this research, the authors showed that chaos can exist in the fractional-order Rössler equation with an order as low as 2.4 and that hyperchaos can exist in the same equation with an order as low as 3.8. In [15], the chaotic behavior of the fractional-order Chen system was examined. The authors determined that chaos exists in the fractional-order Chen system with an order less than 3. In [16], Lu numerically investigated the chaotic behavior of the fractional-order Lü system. A remarkable finding is that the lowest order for this system to exhibit chaos is 0.3; this system is therefore the lowest-order chaotic system among all chaotic systems reported in the literature. More fractional-order systems with chaotic behavior are discussed in detail in [17–19]. However, these studies do not focus much on estimating the parameters of fractional-order chaotic systems.

To date, much work has been made on the parameter estimation of integer-order chaotic systems. Dai et al. [20] used the genetic algorithm (GA) to estimate the parameters of the Lorenz chaotic system. Li et al. [21] utilized a chaotic ant swarm algorithm to identify the parameters of the logistic iteration and Lorenz systems. In [22], differential evolution was used to identify the parameters of the Lorenz system. In [23], the particle swarm optimization (PSO) algorithm was applied to solve the parameter estimation of chaotic systems. Numerical simulation shows that PSO is a feasible approach for the parameter identification of integer-order chaotic systems. Although some progress has been made in the parameter estimation of integer-order chaotic systems, the parameter estimation of fractional-order chaotic systems is more complicated than that of integer-order chaotic systems. Therefore, we put forward QPPSO to solve the complicated problem of parameter estimation of fractional-order chaotic systems. Moreover, QPPSO can also be applied to many other aspects such as 3-d object retrieval and recognition[24], hyperspectral image classification[25,26] and visual-codebook compression[27].

## Problem Description

Consider the following *n*-dimensional fractional-order chaotic system [28]:

$$D^{q}X=F(X,X_{0},\theta ),$$ (1)

where *X* = (*x*_1_, *x*_2_, …, *x_n_*)^*T*^ ∈ *R^n^* denotes the *n*-dimensional state vector of the original system, *X*_0_ represents the system initial state, *q* = (*q*_1_, *q*_2_, …, *q_n_*)^*T*^ ∈ *R^n^* is a set of fractional order of the original system, and *θ* = (*θ*_1_, *θ*_2_, …, *θ*_D_)^T^ ∈ *R^D^* is the value of the original system parameters.

Suppose that the structure of the system is determined in advance, therefore the estimated system can be described as follows:

$$D^{\hat{q}}\hat{X}=F\left(\hat{X},X_{0},\hat{\theta }\right),$$ (2)

where $\hat{X}={\left({\hat{x}}_{1},{\hat{x}}_{2},\ldots ,{\hat{x}}_{n}\right)}^{T}\in R^{n}$ expresses the *n*-dimensional state vector of the estimated system, $\hat{q}={\left({\hat{q}}_{1},{\hat{q}}_{2},\ldots ,{\hat{q}}_{n}\right)}^{T}$ is the estimated order of the system, and $\hat{\theta }={\left({\hat{\theta }}_{1},{\hat{\theta }}_{2},\ldots ,{\hat{\theta }}_{D}\right)}^{T}$ is a set of estimated parameters.

Essentially, the parameter estimation of the fractional-order chaotic system problem considered here involves searching for the optimal parameters $\hat{q}$ and $\hat{\theta }$, the performance index shown in Eq. 3 is minimized.

$$\min J=\frac{1}{M}{\sum_{k=1}^{M}\left\|X_{k}-{\hat{X}}_{k}\right\|}^{2},$$ (3)

where *M* denotes the length of data used for parameter estimation. *X_k_* and ${\hat{X}}_{k}$ (*k* = 1, 2, ..., *M*)denote the state of the original and the estimated systems at time *k*, respectively.

**Figure 1:**
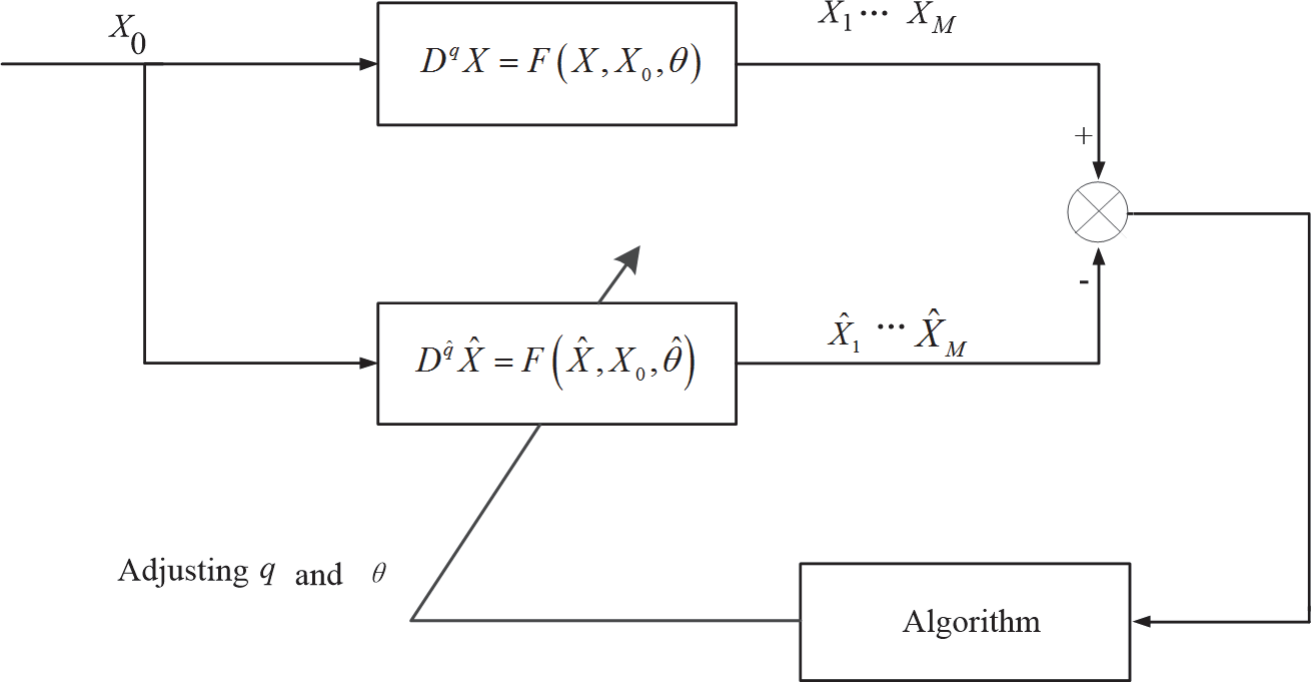
Schematic of fractional-order chaotic system parameter estimation. The parameter estimation for the fractional-order chaotic system can be considered a multidimensional continuous optimization problem, where *q* and *θ* are the decision variables.

Evidently, the parameter estimation for the fractional-order chaotic system can be considered asa multi-dimensional continuous optimization problem, where *q* and *θ* are the decision variables, and *J* is the optimization goal. The principle of parameter estimation for fractional-order chaotic systems in the optimization sense is shown in Fig. 1.

Because of the unstable dynamic behavior of fractional-order chaotic systems, accurate parameters are difficult to obtain. Moreover, traditional optimization methods are difficult to derive in global optimal parameters as many local optima in the landscape of *J*. Therefore, a novel algorithm called QPPSO is proposed to solve the parameter estimation of fractional-order chaotic systems.

## QPPSO

### Quantum parallelism

Quantum parallelism, which enables quantum computers to simultaneously evaluate a function *f*(*x*) for many different values of *x*, is a fundamental feature of numerous quantum algorithms.

**Figure 2:**
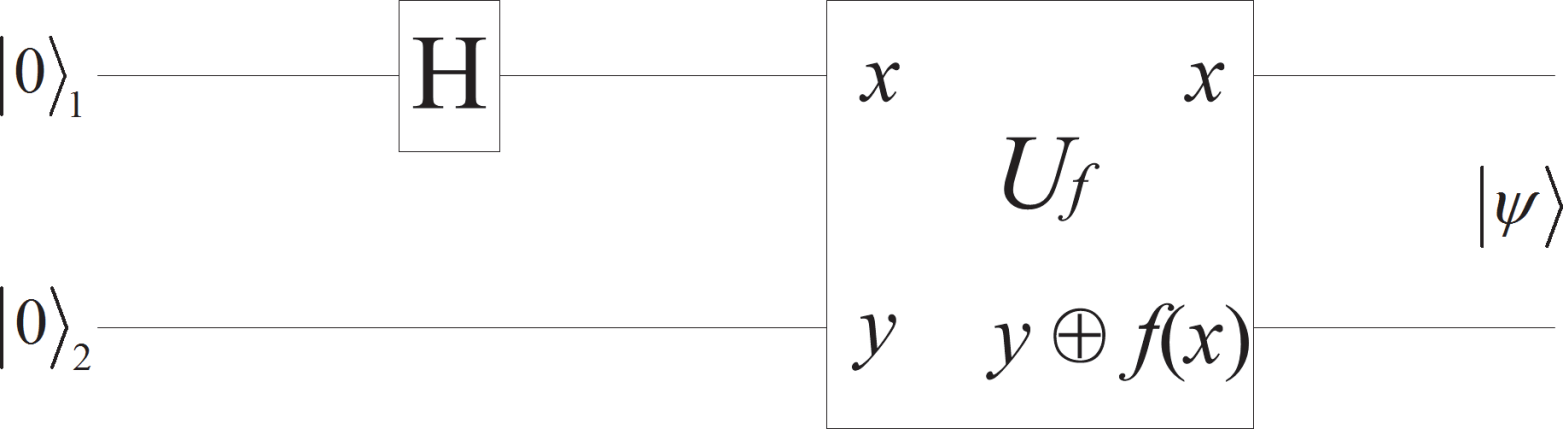
Quantum circuit to simultaneously evaluate *f*(*x*). *U_f_* is the quantum circuit that takes inputs, such as |x,y〉, to |x,y⊕f(x)〉.

Consider the circuit shown in Fig. 2. The circuit applies *U_f_* to an input that is not in the computational basis. The data register is prepared in the superposition $\left(|0\rangle +|1\rangle \right)/\sqrt{2}$, which is created with a Hadamard gate acting on |0〉. The state |ψ〉 is then calculated with *U_f_* as follows [29]:

$$|\psi \rangle =U_{f}H|0,0\rangle =U_{f}\frac{1}{\sqrt{2}}\left(|0,0\rangle +|1,0\rangle \right)=\frac{1}{\sqrt{2}}\left(|0,f\left(0\right)\rangle +|1,f\left(1\right)\rangle \right)$$ (4)

where |⋅〉 is called the Dirac notation and it is the standard notation for states in quantum mechanics, *H* is the Hadamard gate, and *U*_f_ is the quantum circuit that takes inputs, such as |x,y〉, to |x,y⊕f(x)〉.

In this study, a single *f*(*x*) circuit is used to simultaneously evaluate the function for multiple values of *x* by utilizing the capability of a quantum computer to superposition different states. This procedure can easily be generalized to functions on an arbitrary number of bits by using a general operation known as the Hadamard transform, or sometimes, the Walsh–Hadamard transform. The result of initially conducting the Hadamard transform on *m* Q-bits in |0〉 state is expressed as follows:

$$\begin{array}{cc} |\psi \rangle & =H^{⊗m}{|0\rangle }^{⊗m}=H⊗H⊗\cdots ⊗H|00\cdots 0\rangle \\ & =\frac{1}{\sqrt{2}}\left(|0\rangle +|1\rangle \right)⊗\frac{1}{\sqrt{2}}\left(|0\rangle +|1\rangle \right)⊗\cdots ⊗\frac{1}{\sqrt{2}}\left(|0\rangle +|1\rangle \right)\\ & =\frac{1}{\sqrt{2^{m}}}\left(|00\cdots 0\rangle +|00\cdots 1\rangle \right)+\cdots +\left(|11\cdots 1\rangle \right)\\ & =\frac{1}{\sqrt{2^{m}}}\sum_{x=0}^{2^{m}-1}|x\rangle \end{array},$$ (5)

where $H^{⊗m}$ is the *m* times inner product of Hadamard, and ${|0\rangle }^{⊗m}$ is the *m* times inner product of |0〉. In Eq. 5, the sum is over all possible values of *x*, that is, the Hadamard transform produces an equal superposition of all computational basis states. Moreover, the Hadamard transform efficiently performs this process and thus produces a superposition of 2^*m*^ states by using *m* gates only.

### Quantum parallel particle swarm optimization algorithm

In this section, we introduce the QPPSO optimization algorithm as follows:

#### Quantum encoding

The Q-bit is the smallest unit of information, which can be expressed as follows:

|φ〉=cosθ|0〉+sinθ|1〉. (6)

**Figure 3:**
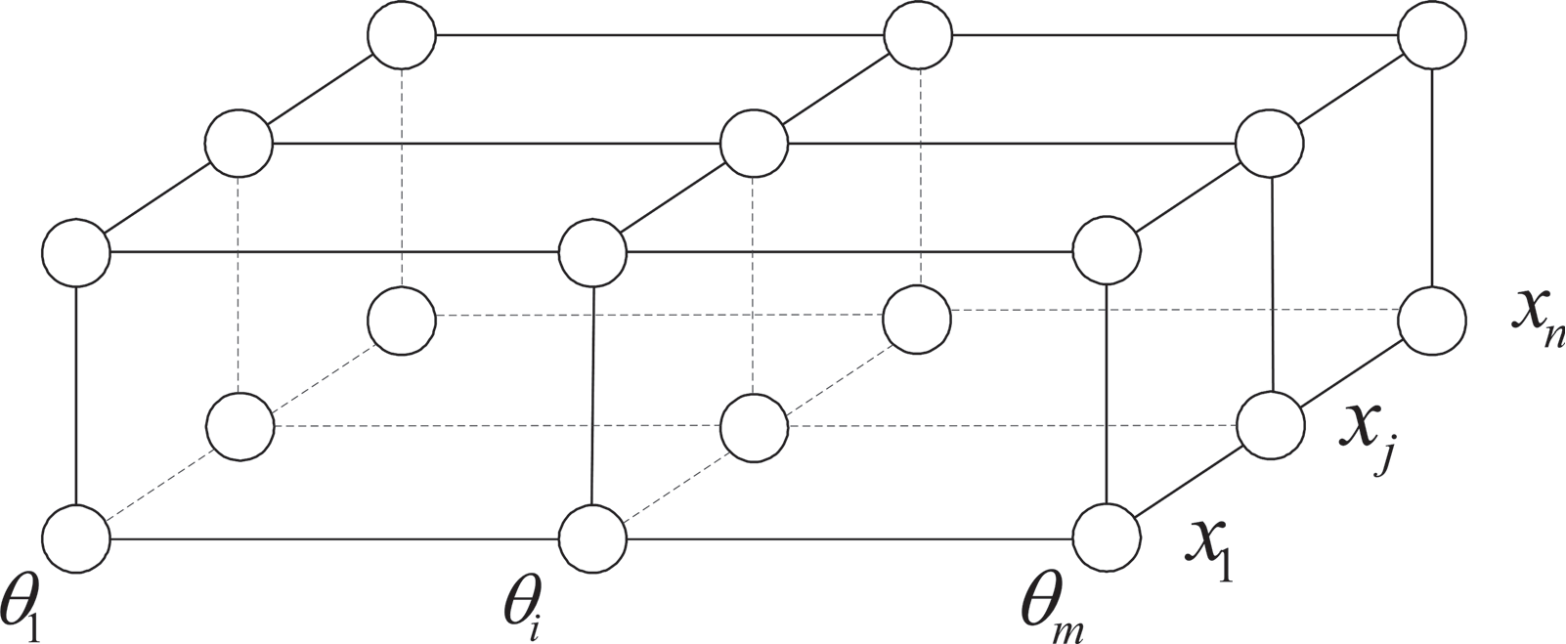
Quantum encoding of QPPSO. For an *n* dimensional space, each basis of this space can be divided into 2^*m*^ states with *m* gates only.

Therefore, the Q-bit can be coded as $\left[\begin{array}{c} \cos\theta \\ \sin\theta \end{array}\right]$, where cos*θ* or sin*θ* just represents a probability amplitude. However, in QPPSO, cos*θ* or sin*θ* is no longer the probability amplitude but is a certain value. The quantum encoding of QPPSO is shown in Fig. 3.

In Fig. 3, an arbitrary *x*_j_ can be expressed as a string of *m* Q-bits shown as follows:

$$|x_{j}\rangle =\left[\begin{array}{lllll} \cos\theta_{1j} & \cdots & \cos\theta_{ij} & \cdots & \cos\theta_{mj}\\ \sin\theta_{1j} & \cdots & \sin\theta_{ij} & \cdots & \sin\theta_{mj} \end{array}\right]$$ (7)

where *θ_ij_* = 2*π* × *rand*; *i* = 1, 2, …, *m*; *j* = 1, 2, …, *n*. *rand* is a random number between zero and one.

Therefore, the tensor product of $|x_{i}\rangle$ with itself can be expressed as follows:

$$\begin{array}{l} |{\text{A}}_{j}\rangle =|x_{1j}\rangle ⊗|x_{2j}\rangle \cdots ⊗|x_{mj}\rangle \\ =\left[\begin{array}{c} \cos\theta_{1j}\\ \sin\theta_{1j} \end{array}\right]⊗\left[\begin{array}{c} \cos\theta_{2j}\\ \sin\theta_{2j} \end{array}\right]\cdots ⊗\left[\begin{array}{c} \cos\theta_{mj}\\ \sin\theta_{mj} \end{array}\right]\\ =\left[\begin{array}{c} \cos\theta_{1j}\times \cos\theta_{2j}\times \cdots \times \cos\theta_{mj}\\ \cos\theta_{1j}\times \cos\theta_{2j}\times \cdots \times \sin\theta_{mj}\\ \cdots \\ \sin\theta_{1j}\times \sin\theta_{2j}\times \cdots \times \sin\theta_{mj} \end{array}\right]\text{=}\left[\begin{array}{c} A_{j1}\\ A_{j2}\\ \cdots \\ A_{j2^{m}} \end{array}\right] \end{array}$$ (8)

**Figure 4:**
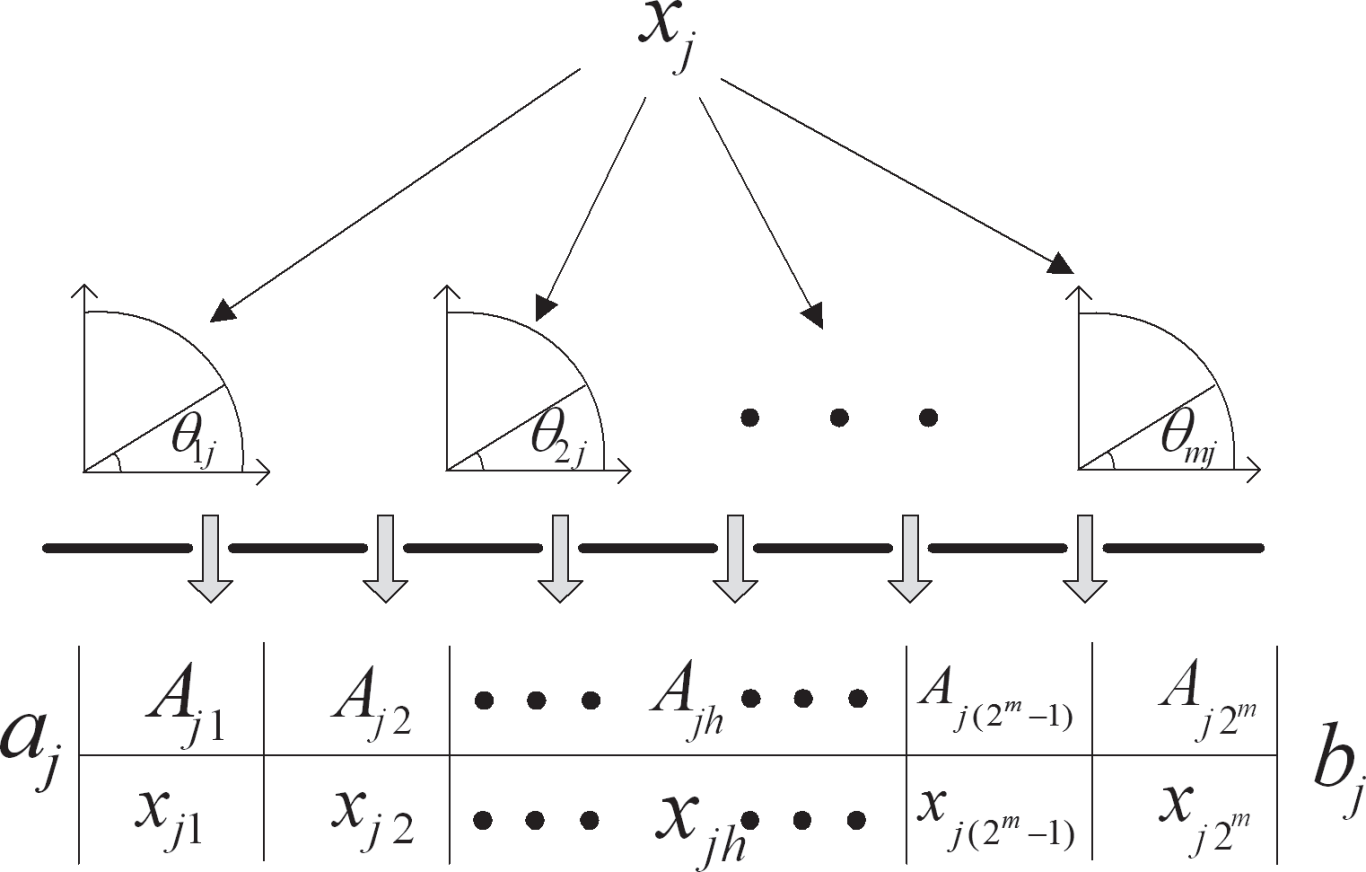
Division of the solution space for *x_j_*. An arbitrary *x_j_* is set a value between (*a*_j_, *b*_j_), and it is divided into 2^m^ subset by quantum parallelism.

For an *n*-dimensional space, each basis in the space has a value range. For simplicity, all the bases are set a value among (*a_j_*, *b_j_*). The division of the solution space is shown in Fig. 4. In Fig. 4, *x_jh_* can be expressed as follows:

$$x_{jh}=\frac{A_{{}^{jh}}+1}{2}\frac{b_{j}-a_{j}}{2^{m}}h,$$ (9)

where *h* = 1, 2, …, 2^*m*^. Based on subpopulation parallel computing, the algorithm running rate is exponentially accelerated. Moreover, individuals belong to different subspaces, so premature phenomena can be efficiently prevented.

#### Quantum state update

In quantum space, the equations in traditional PSO are inapplicable because of the uncertainty relation between the coordinate and the momentum. To restrain the behavior of particles in quantum space, a formula that consists of the current rotation angle, the individual optimal quantum rotation angle, and the global optimal quantum rotation angle is proposed and expressed as follows:

$$\theta_{ijk}\left(t+1\right)=\frac{c_{1}r_{1}\theta_{P_{ijk}}\left(t\right)+c_{2}r_{2}\theta_{G_{i}{}_{j}}\left(t\right)}{\left(c_{1}r_{1}+c_{2}r_{2}\right)}\pm w\cdot \ln\left[1/u_{ijk}\left(t\right)\right]\left|\frac{1}{L}\sum_{k=1}^{L}\theta_{P_{ijk}}\left(t\right)-\theta_{ijk}\left(t\right)\right|,$$ (10)

where *k* = 1, 2, …, *L, L* is the population size of QPPSO, *c*_1_ and *c*_2_ are constants, *w* is the inertia weight, *r*_1_, *r*_2_, and *u_ijk_*(*t*) are the random numbers between zero and one, and *t* is the current iteration number.

#### Structure of QPPSO

**Figure 5:**
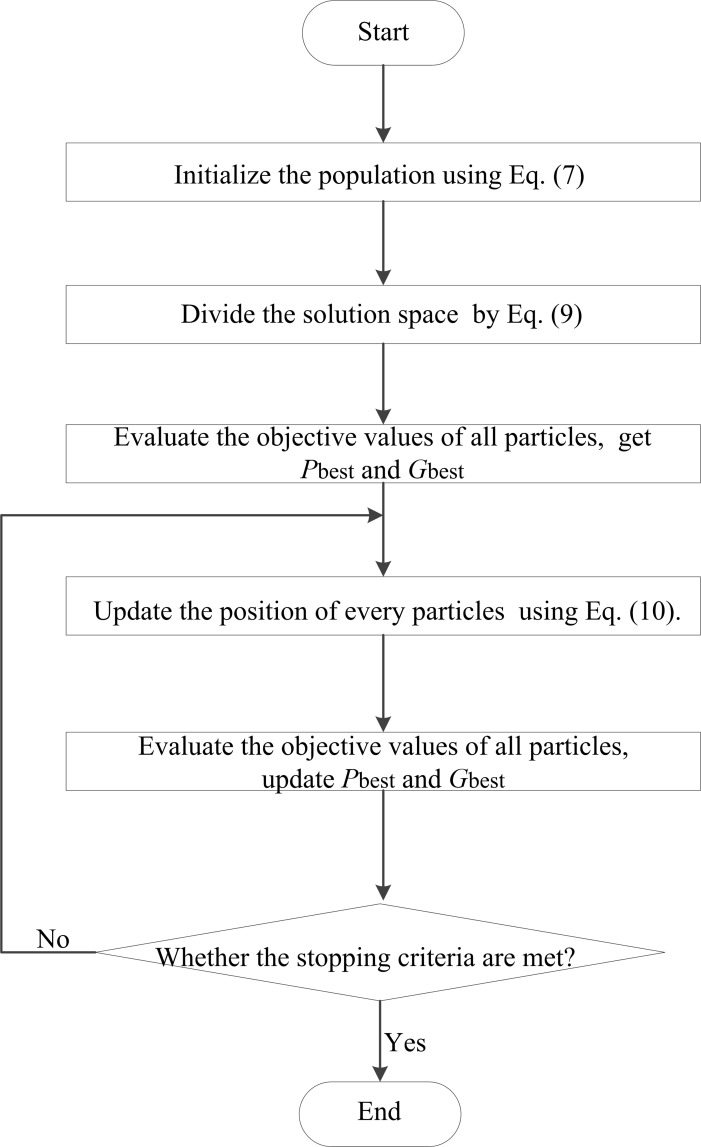
Flowchart of the QPPSO algorithm. The procedure of the QPPSO algorithm.

The procedure of the QPPSO algorithm is summarized in the following section, and the flowchart of QPPSO is shown in Fig. 5.

Step 1: Initialize the parameter sets of the algorithm. The population is initialized by quantum encoding in Eq. (7), and the solution space is decomposed by Eq. (9).
Step 2: The objective values of all particles are evaluated. We set the *p_best_* of each particle and its objective value equal to the current position of the particle and to its objective value, and then set *g_best_* and its objective value equal to the position of the particle and to the objective value of the best initial particle.
Step 3: The position of every particle is updated by Eq. (10).
Step 4: The objective values of all particles are evaluated.
Step 5: The current objective value of each particle is compared with the objective value of its *p_best_*. If the current value is better, then *p_best_* and its objective value are updated with the current position.
Step 6: The best particle of the current population with the best objective value is determined. If the objective value is better than that of *g_best_*, then *g_best_* and its objective value are updated with the position and objective value of the current best particle.
Step 7: If the stopping criteria are met, we output *g_best_* and its objective value; otherwise, we go back to Step 3.

## Simulation and Comparisons

### Typical fractional-order chaotic systems

In this section, numerical simulation and comparisons are conducted on the basis of some typical fractional-order chaotic systems, including the fractional-order Chen system, fractional-order Lorenz system, fractional-order Rössler system, and fractional-order Lü system.

- The fractional-order Chen chaotic system can be expressed as follows:
  $$\left\{\begin{array}{l} \frac{d^{q_{1}}x_{1}}{\text{d}t^{q_{1}}}=a\cdot \left(x_{2}-x_{1}\right)\\ \frac{d^{q_{2}}x_{2}}{\text{d}t^{q_{2}}}=\left(c-a\right)\cdot x_{1}-x_{1}\cdot x_{3}+c\cdot x_{2}\\ \frac{d^{q_{3}}x_{3}}{\text{d}t^{q_{3}}}=x_{1}\cdot x_{2}-b\cdot x_{3} \end{array}\right.,$$ (11)
  where *a, b, c, q*_1_, *q*_2_, and *q*_3_ are the unknown constant parameters of the fractional-order chaotic systems that should be estimated. When *a* = 35, *b* = 3, *c* = 28, *q*_1_ = 0.93, *q*_2_ = 0.9, and *q*_3_ = 0.88, this system exhibits a chaotic dynamical behavior.
- The fractional-order Lorenz chaotic system is expressed as follows:
  $$\left\{\begin{array}{l} \frac{d^{q_{1}}x_{1}}{\text{d}t^{q_{1}}}=a\cdot \left(x_{2}-x_{1}\right)\\ \frac{d^{q_{2}}x_{2}}{\text{d}t^{q_{2}}}=\left(c-a\right)\cdot x_{1}-x_{1}\cdot x_{3}+c\cdot x_{2}\\ \frac{d^{q_{3}}x_{3}}{\text{d}t^{q_{3}}}=x_{1}\cdot x_{2}-b\cdot x_{3} \end{array}\right..$$ (12)
  The system is in a chaotic state when *a* = 10, *b* = 28, *c* = 8/3, *q*_1_ = 0.993, *q*_2_ = 0.993, and *q*_3_ = 0.993.
- The fractional-order Rössler chaotic system can be expressed as follows:
  $$\left\{\begin{array}{l} \frac{d^{q_{1}}x_{1}}{\text{d}t^{q_{1}}}=-\left(x_{2}+x_{3}\right)\\ \frac{d^{q_{2}}x_{2}}{\text{d}t^{q_{2}}}=x_{1}+a\cdot x_{2}\\ \frac{d^{q_{3}}x_{3}}{\text{d}t^{q_{3}}}=b+x_{3}\left(x_{1}-c\right) \end{array}\right..$$ (13)
  The system is in a chaotic state when *a* = 0.5, *b* = 0.2, *c* = 10, *q*_1_ = 0.9, *q*_2_ = 0.85, and *q*_3_ = 0.95.
- The fractional-order Lü chaotic system can be expressed as follows:
  $$\left\{\begin{array}{l} \frac{d^{q_{1}}x_{1}}{\text{d}t^{q_{1}}}=a\left(x_{2}-x_{1}\right)\\ \frac{d^{q_{2}}x_{2}}{\text{d}t^{q_{2}}}=-x_{1}\cdot x_{3}+c\cdot x_{2}\\ \frac{d^{q_{3}}x_{3}}{\text{d}t^{q_{3}}}=x_{1}\cdot x_{2}-b\cdot x_{3} \end{array}\right..$$ (14)
  The system is in a chaotic state when *a* = 36, *b* = 3, *c* = 20, *q*_1_ = 0.985, *q*_2_ = 0.99, and *q*_3_ = 0.98.

**Figure 6:**
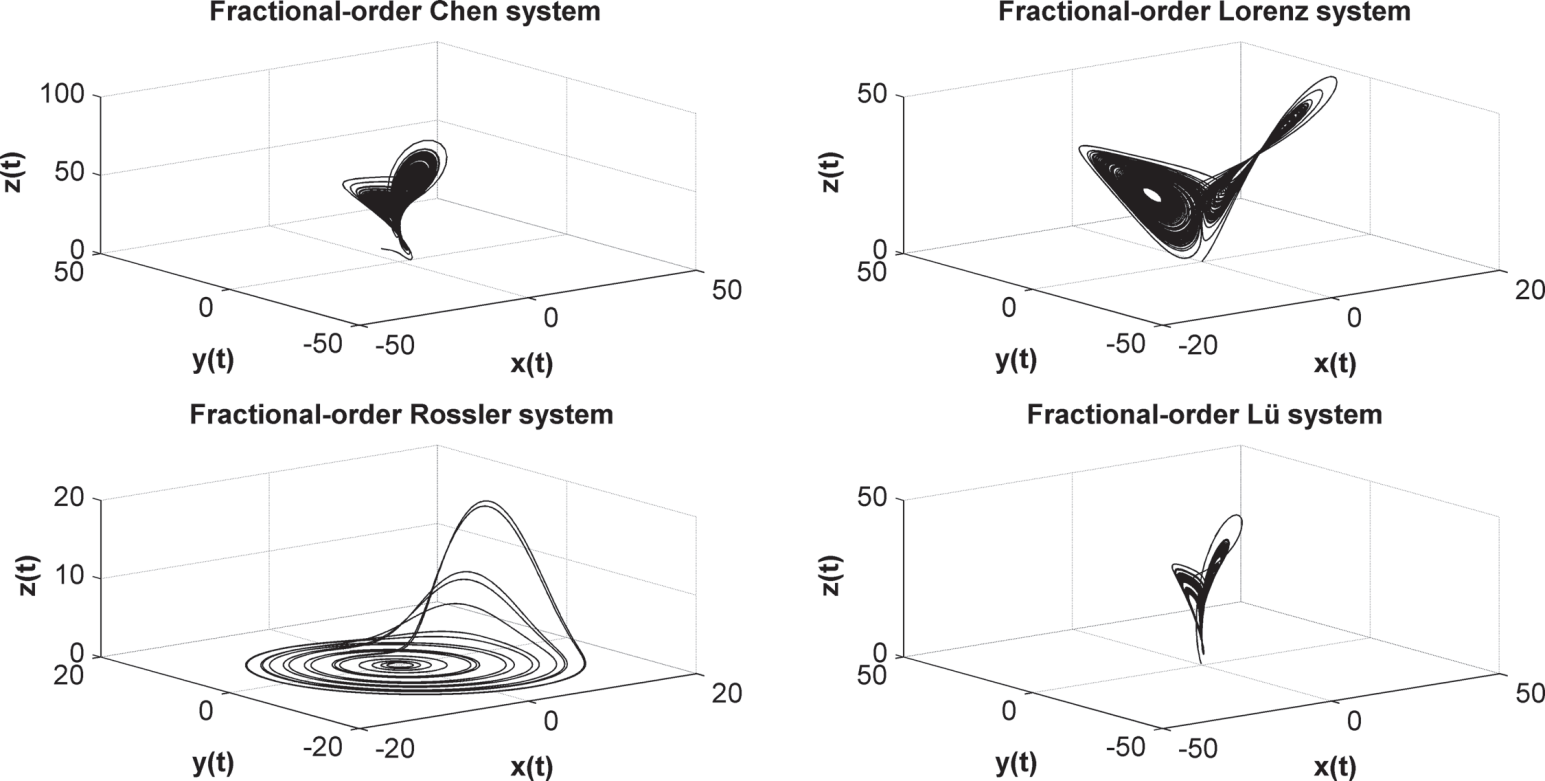
Chaotic behavior of four typical fractional-order chaotic systems. (a) Chaotic behavior of the fractional-order Chen system when a = 35, b = 3, c = 28, q_1_ = 0.93, q_2_ = 0.9, and q_3_ = 0.88; (b) Chaotic behavior of the fractional-order Lorzen system when a = 10, b = 28, c = 8/3, q_1_ = 0.993, q_2_ = 0.993, and q_3_ = 0.993; (c) Chaotic behavior of the fractional-order Rössler system when a = 0.5, b = 0.2, c = 10, q_1_ = 0.9, q_2_ = 0.85, and q_3_ = 0.95; (d) Chaotic behavior of the fractional-order Lü system when a = 36, b = 3, c = 20, q_1_ = 0.985, q_2_ = 0.99, and q_3_ = 0.98.

For the previously discussed systems, we solve all parameters by using the numerical algorithm derived from the G-L definition of fractional derivatives to obtain the state variables *x, y, z*. Numerical results show that these systems are chaotic, and their chaotic behavior is shown in Fig. 6.

### Simulations on the systems

In our simulation, all previously discussed systems freely evolve from random initial states. After a period of the transient process, a state vector is selected as the initial state *X*_0_ for parameter estimation (Fig. 1). The sampling time is *h* = 0.01, and the total number of states to calculate *J* is set as 100. We compare our QPPSO with GA and PSO. For a fair comparison, the maximum generation number and the searching range of the parameters are the same in all algorithms. That is, the maximum generation number is set as 1,000, and the population size is set as 80. The searching spaces of the parameters are shown in Table 1. The other settings for the parameters of the algorithms are as follows. For the GA method, the crossover rate is set as 0.9, and the mutation probability is set as *Pm* = 0.1. For the PSO method, according to Clerc’s stagnation analysis [30], the inertia weight is set as *ω* = 1/(2 × log(2)), and two acceleration coefficients are set as *c*_1_ = *c*_2_ = 0.5 + log(2). The probability threshold for random topology is set as *Pr* = 1 − (1 − 1/*s*)^3^, where *s* is the population size, and *Pr* is used to determine the proportion of local informants to the entire population. For QPPSO, we also set *c*_1_ = *c*_2_ = 0.5 + log(2) and the inertia weight *ω* = 1/(2 × log(2)).

**Table 1:** Upper/lower bounds for the parameters of fractional-order chaotic systems.

**System**	**Bounds**	***a***	***b***	***c***	***q*_1_**	***q*_2_**	***q*_3_**
Chen	Lower bound	30	2	25	0.85	0.85	0.85
	Upper bound	40	5	30	1	1	1
							
Lorenz	Lower bound	5	25	1	0.9	0.9	0.9
	Upper bound	15	30	5	1	1	1
							
Rössler	Lower bound	0	0	5	0.8	0.8	0.8
	Upper bound	1	1	15	1	1	1
Lü	Lower bound	30	1	15	0.9	0.9	0.9
	Upper bound	40	5	25	1	1	1

For the simulation, the search space for the parameters is given in the table.

Table 2 shows the mean of the objective function values, the standard deviation, and the best objective value of 50 independent runs. Table 3 shows the estimation values for the parameters of the chaotic systems.

**Table 2:** Objective function value.

System	Method	Mean±std	Min
Chen	GA	1.4829e-02±2.7205e-02	3.4212e-03
	PSO	2.5895e-04±1.173e-04	5.7923e-05
	QPPSO	4.5487e-05±3.5697e-05	4.7749e-06
			
Lorenz	GA	2.0363e-03±5.1583e-03	3.5258e-04
	PSO	1.4046e-04±1.3289e-04	8.7529e-05
	QPPSO	9.6230e-06±2.2486e-06	1.4657 e-06
			
Rössler	GA	8.7892e-05±2.3452e-06	5.3245e-06
	PSO	5.4517e-07±8.5761e-07	1.7434e-07
	QPPSO	8.7813e-11±1.0903e-10	1.1146e-11
			
Lü	GA	8.5438e-03±2.14234e-04	3.1457 e-03
	PSO	4.2567e-04±8.2679e-05	1.7754e-04
	QPPSO	1.3644e-04±4.9176e-05	4.9304e-05

The mean of the objective function values, standard deviation, and best objective value of 50 independent runs are computed by GA, PSO and QPPSO

**Table 3:** Estimation values for the parameters of fractional-order chaotic systems.

**System**	**Method**	***â***	$\hat{b}$	$\hat{c}$	${\hat{q}}_{1}$	${\hat{q}}_{2}$	${\hat{q}}_{3}$
		**Mean±std**	**Mean±std**	**Mean±std**	**Mean±std**	**Mean±std**	**Mean±std**
Chen	GA	33.5267±1.5782	3.1092±0.1586	27.3883±0.1258	0.9198±0.0086	0.8925±0.0009	0.8884±0.0009
	PSO	34.5889±0.8952	3.0153±0.1025	27.8798±0.0952	0.9271±0.0085	0.8982±0.0008	0.8814±0.0005
	QPPSO	35.1926±0.1729	2.9928±0.0061	28.0560±0.0509	0.9314±0.0012	0.9008±0.0008	0.8793±0.0006
	True values	35	3	28	0.93	0.9	0.88
Lorenz	GA	9.8662±1.0037	28.0546±0.9331	2.7556±0.1468	0.9812±0.0184	0.9964±0.1012	0.9970±0.0085
	PSO	10.3961±0.6175	27.6073±0.7953	2.7119±0.2682	0.9966±0.0067	0.9936±0.0038	0.9941±0.0024
	QPPSO	9.9084±0.3482	28.1636±0.2568	2.6528±0.2568	0.9891±0.0065	0.9953±0.0024	0.9914±0.0031
	True values	10	28	8/3	0.993	0.993	0.993
Rössler	GA	0.3311±0.3516	0.4944±04538	13.1179±2.5468	0.9200±0.2489	0.8835±0.3543	0.9600±0.1524
	PSO	0.3528±0.2523	0.4354±0.1259	12.7304±1.2584	0.9172±0.1537	0.8796±0.2564	0.9042±0.4187
	QPPSO	0.4996±0.0100	0.2249±0.0831	11.2479±0.5237	0.9000±0.0001	0.8501±0.0034	0.9636±0.0135
	True values	0.5	0.2	10	0.9	0.85	0.95
Lü	GA	34.7924±1.4385	3.0126±0.0084	20.0392±0.0851	0.9722±0.0085	0.9896±0.0014	0.9845±0.0012
	PSO	36.7016±0.5844	3.1996±0.0348	20.4791±0.1259	0.9823±0.0023	1.0000±0.0025	0.9912±0.0009
	QPPSO	36.2826±0.1710	2.9890±0.0069	20.0516±0.0330	0.9870±0.0012	0.9911±0.0007	0.9789±0.0007
	True values	36	3	20	0.985	0.99	0.98

The mean and standard deviation of estimation values are computed by GA, PSO and QPPSO, and they are compared with the true values

**Figure 7:**
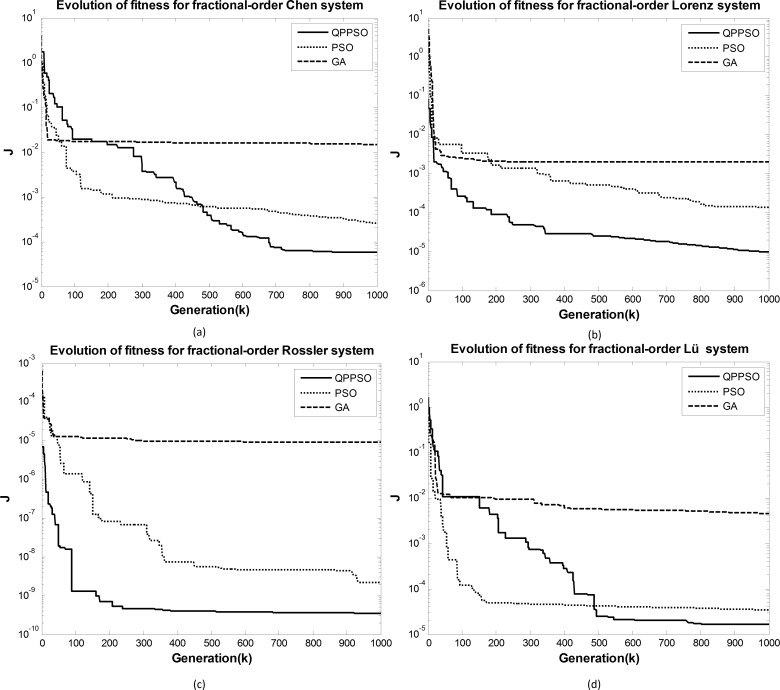
Convergence graph of the objective function. (a) Convergence graph of the fractional-order Chen system, (b) Convergence graph of the fractional-order Lorzen system, (c) Convergence graph of the fractional-order Rössler system, (d) Convergence graph of the fractional-order Lü system.

The results shown in the tables indicate that QPPSO has better performance than the GA and PSO methods in the parameter estimation of fractional-order chaotic systems. This conclusion can also be obtained from the convergence curves of the objective function for the different methods displayed in Fig. 7. In this figure, the logarithmic scale is used for the y-axis for convenience in plotting the data.

For the fractional-order Chen and Lü chaotic systems, the converging speed for QPPSO is slower than those for the GA and PSO methods in the initial period of evolution. However, QPPSO converges more quickly than the GA and PSO methods during the later period of evolution. The estimation values for the parameters in QPPSO are more accurate than those for the parameters in the GA and PSO methods (Table 3).

For the fractional-order Lorenz and Rössler chaotic systems, the converging speed for QPPSO is faster than those for the GA and PSO methods during all periods of evolution. Table 3 also shows that the estimation values for the parameters in QPPSO are more accurate than those for the parameters in the GA and PSO methods.

**Figure 8:**
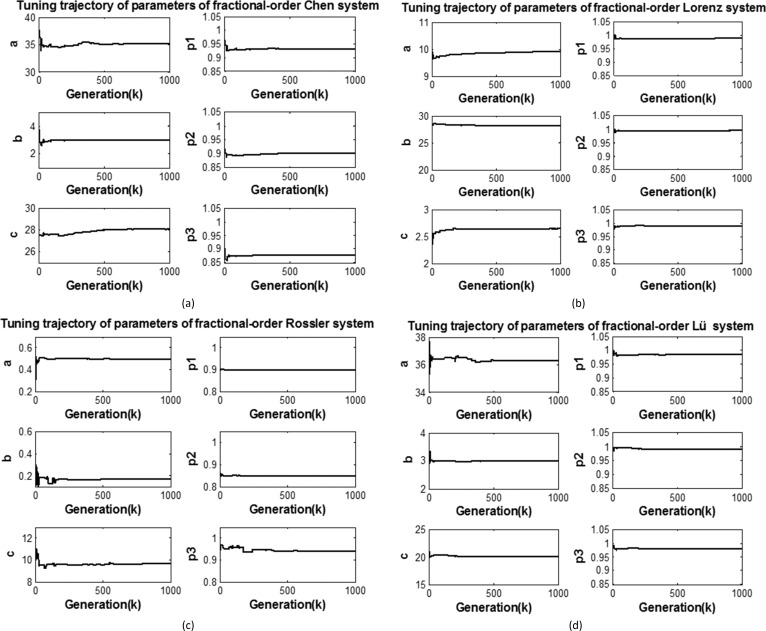
Tuning trajectories of the parameters of fractional-order chaotic systems by the QPPSO method. (a) Tuning trajectory of the parameters of the fractional-order Chen system, (b) Tuning trajectory of the parameters of the fractional-order Lorzen system, (c) Tuning trajectory of the parameters of the fractional-order Rössler system, (d) Tuning trajectory of the parameters of the fractional-order Lü system.

Fig. 8 shows one typical run of the tuning trajectories of the parameters of fractional-order chaotic systems with respect to the number of generations by the QPPSO method. Less than 500 iterations are needed for the parameters to reach the steady state and converge to the actual parameters. This result shows the effectiveness and feasibility of using QPPSO to estimate the parameters of fractional-order chaotic systems.

From the above results, we can conclude that the calculation method of the introduced quantum computing parallel characteristic can improve the ergodicity of the algorithm and then increase the ability of global convergence of the algorithm. Moreover, the quantum states updated by Eq. (10) can make the convergence speed of QPPSO faster than that of PSO because the speed limited equation is no longer suitable in quantum space. The above results also show the effectiveness and efficiency of QPPSO.

## Conclusion

This study proposed a new QPPSO algorithm that was applied to solve the unknown parameter estimation of the fractional-order chaotic system. The parallel characteristic of quantum computing is used in QPPSO. This characteristic causes the calculation of each generation to exponentially increase. The behavior of particles in quantum space is restrained by the quantum evolution equation, which consists of the current rotation angle, individual optimal quantum rotation angle, and global optimal quantum rotation angle. Numerical simulation based on several typical fractional-order systems and comparisons with some typical existing algorithms demonstrate the effectiveness and efficiency of the proposed algorithm. Future work should develop QPPSO-based approaches more effective and adaptive than current ones and apply the algorithm to other systems.
